# Disseminated cryptococcosis with cutaneous involvement in an immunocompetent patient*

**DOI:** 10.1590/abd1806-4841.20165478

**Published:** 2016

**Authors:** Gabriely Lessa Sacht, Alexandre Moretti de Lima, Yuri Chiarelli Perdomo, Rafaela Suguimoto Boigues, Luiz Carlos Takita, Günter Hans Filho

**Affiliations:** 1 Universidade Federal de Mato Grosso do Sul (UFMS) – Campo Grande (MS), Brasil

**Keywords:** Cryptococcosis, *Cryptococcus* gattii, Yeasts

## Abstract

Cryptococcosis is a fungal infection of opportunistic behavior that is unusual in immunocompetent patients. We report a rare case of disseminated cryptococcosis with cutaneous involvement in an immunocompetent individual. During hospitalization, *Cryptococcus* gattii was isolated from skin lesions, lung and spinal fluid. The diagnosis of disseminated *cryptococcosis* was confirmed and treatment was established. The patient showed improvement. Due to the probable clinical severity of the disease and the possibility that skin lesions may be the first manifestation of this illness, prompt diagnosis must be established and treatment provided.

A male patient, mason, 33-years-old, reported the presence of papulonodular lesions on his face and dorsum over the past 30 days (Figure 1). His symptoms progressed with the development of headache, dizziness, malaise, and fever. Complete blood count and kidney and liver function tests were normal. HIV, HTLV, viral hepatitis and VDRL serological tests were also normal. Histopathological examination of the skin lesion revealed mucus and numerous *Cryptococci* on the dermis (Figure 2). Direct mycological examination and culture showed yeast suggestive of *Cryptococcus ssp* (Figure 3). Specific biochemical tests indicated *Cryptococcus gattii* growth. Spinal cord fluid showed lymphocytic pleocytosis and increased protein and reduced glucose levels. Direct assay and culture were positive for *C. gattii.* Computed tomography of the chest and bronchoscopy showed a lesion in the lower lobe of his left lung (Figure 4). An assay of *Cryptococcus ssp*. in the bronchial lavage fluid was positive. We prescribed amphotericin B deoxycholate (50mg/day) and fluconazole (450 mg every 12 hours for four weeks and maintenance dose of 300mg/week for eight weeks). The patient showed improvement of skin lesions and neurological and pulmonary symptoms.

**Figure 1:**
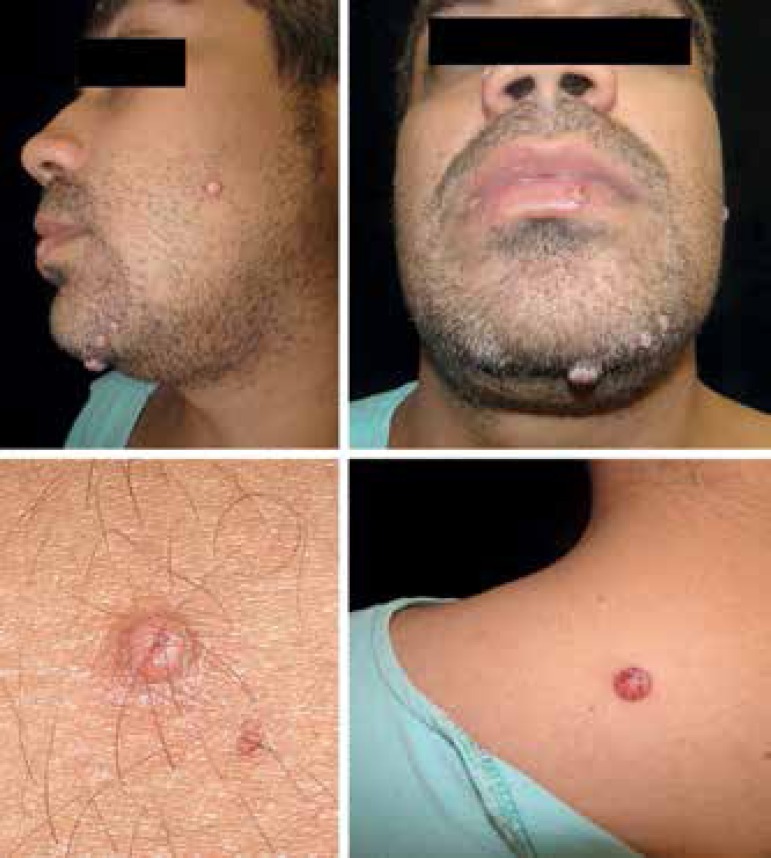
Papulonodular lesions, some showing hematic crusts and umbilicated center, on the face and upper dorsum

**Figure 2:**
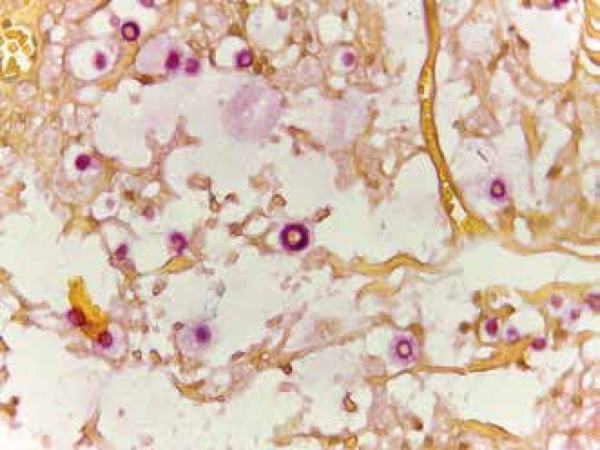
Presence of rounded structures compatible with Cryptococcus ssp.(Mucicarmine stain, 400x)

**Figure 3:**
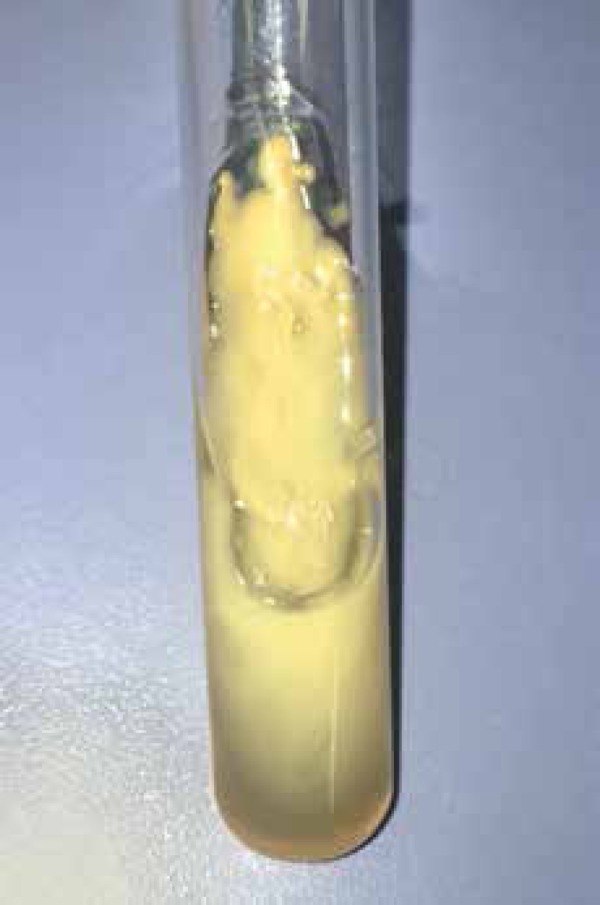
Milky colony growth, similar to dripping candle wax, in Sabouraud agar, suggestive of *Cryptococcus* ssp.

**Figure 4:**
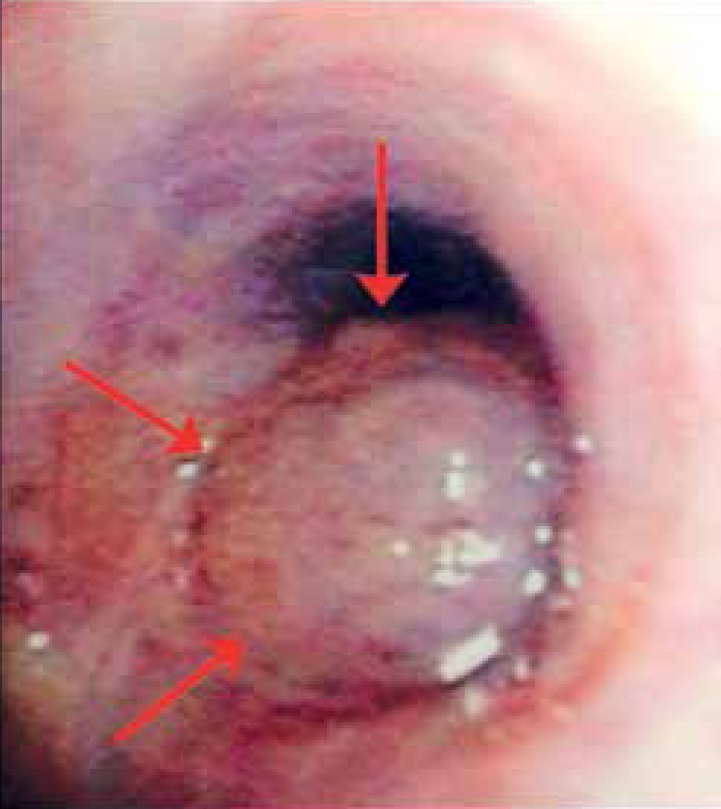
Bronchoscopy showing a tumor lesion located in the lower lobe of the left lung.

## DISCUSSION

Cryptococcosis in immunocompetent patients is endemic in tropical and subtropical areas. It is predominantly caused by C. *gattii* ^1-3^ This fungus has been associated with plant litter of *Eucalyptus camaldulensis.* This does not represent its natural habitat, which reveals different geographic patterns of the occurrence of fungus-tree-decaying wood ^2,4,5^ Our patient reported wood handling in recent months. Cutaneous lesions of cryptococcosis may be the first manifestation of the systemic form of the disease, even in immunocompetent patients. In the disseminated forms of the disease, skin lesions are polymorphic. ^3.6^ When lesions resemble those of molluscum contagiosum, we should consider the diagnosis of cryptococcosis, especially in immunocompromised patients.
